# King Solomon's Physic

**Published:** 1887-09-17

**Authors:** 


### The Doctor's Pulpit.

### KING SOLOMON'S PHYSIC.

The public has lately heard a good deal about King Solomon's mines; perhaps it may not be disinclined to hear a little about his physic. Of course we would not for a moment indulge the idea that "physic" is so popular and tempting a subject as "mines"; but "every man to his trade"; and as the fates have ordained that physic shall be the subject-matter both of our preaching and our practice, we make bold to ask readers for a brief space to "lend us their ears." Solomon clearly was a believer in physic; he was not one of those very wise people who have "no faith in doctors." If anybody disputes that proposition he is referred to the world-famous "Proverbs," to certain utterances preserved in other ancient Jewish literature, and to oral tradition. If with such convincing evidence as will be found in those various sources of information the sceptic is not convinced, we can only express the conviction that he is very difficult to convince.

King Solomon was undoubtedly a great man, a conspicuous figure among the ancients, and is by no means forgotten by the moderns. We do not, therefore, propose to give a mere catalogue of his remedies for various diseases to gratify curiosity. That would be treating him with scant courtesy, and gaining little personal advantage from his medical lore. Many of his single prescriptions are as worthy of a lengthy article as Burns's pudding—the famous haggis—was worthy of a "grace" "as lang's my airm"; and one such prescription we shall here give, with such a measure of expansion and enforcement as may be profitable to all who read it.

The prescription, we may premise, does not begin with the cabalistic ℞ beloved of modern physicians. It is rather in the form of a broad general principle, which may be understood of all mankind, and can be tried and tested by everybody without a preliminary visit to the chemist's shop, or the cheaper but possibly less trustworthy stores. Perhaps, however, it may be considered to labour under a disadvantage, in that the prescriber, so far as we are aware, never demanded any fee for it, and certainly does not do so now. That indeed will probably be regarded as a fatal objection by many, especially of the gentler sex, who usually value everything in strict proportion to its price. By them, therefore, it may be feared that King Solomon's prescription will be held in small esteem, in comparison with that of their favourite Æsculapius, who dispenses gracious smiles along with the latest form of medical wisdom at the substantial but not unreasonable rate of two guineas for each consultation.

Here, then, is the prescription, and it is a perfect model of simplicity, brevity, and comprehensiveness,—"*A merry heart doeth good like a medicine.*" The late Sir Robert Christison, who for many years was Physician to the Queen in Scotland, was a great experimenter with drugs, and for a long time the person on whom he made the earliest experiment with a new remedy was himself. This practising upon himself gave him a knowledge of the remedy, and a confidence in it which no other method of experimentation could possibly have supplied. King Solomon, we are perfectly certain, was a man who made personal experiments. He was a true Bacon long before Bacon was born. Anyone who is accustomed to read books with understanding easily discovers that by far the larger number of Solomon's utterances bear the unmistakable "hall-mark" of "experience." It is as certain as anything can be that when he spoke of merriment, brightness, of soul, joyfulness, as a medicament, a substitute for the "pill and the physic-bottle," he spoke of what he had tried upon himself and upon other people many a time. He knew perfectly well what he was talking about, just as he did when he added the other part of the proverb,—"a broken spirit drieth the bones." It is amazing to us, as it must be to many people, what little store the men of one generation set by the experience of the men of a past generation. Nothing can be more foolish. Experience can never be without value, even if it be the experience of a fool; whilst the experience of a clear-eyed, observant, and thoughtful man is beyond all price. Any man who has travelled through the wilds of Africa must know more about the Africans than the cleverest Board-School master who teaches geography to his boys. But an able man who has spent years on that continent cannot but possess stores of the most valuable knowledge, such as all the Geographical Societies in the world could never acquire merely by staying at home. The man who despises experience is hopeless, although he be a Fellow of fifty Royal Societies.

To come back to Solomon. He had tried his recipe, we are persuaded, in every form; and being convinced of its character as the purest medical gold, he put it into the fewest possible words, and sent it forth on its mission into the great, wide world. Here it is to-day, fresh and untarnished; and if you ring it on the counter of nineteenth century science and experience, it rings as true and as honest as it did the very day on which it was coined in Solomon's intellectual mint. "A merry heart doeth good like a medicine." It is undeniable that medical practice must be varied with varying times and circumstances, and this remedy must be employed at proper seasons. What is good for summer may not be best for winter. What is suitable for the country may be very unsuitable for the town; and a medicine which cured the robust and strong-nerved men and women of Queen Bess's period might kill the more nervous and sensitive subjects of Queen Victoria. The able physician is he who understands the men and women of the time in which he lives, and out of the stores, "new and old," of his own and the world's experience, draws remedies which may best meet the necessities of the hour.

Such a remedy is this of Solomon's for the present time. The world, especially the world of London and other large towns, is terribly serious —is it not even sad? What is the use of the universal sadness: what the necessity for it? Providence is not dead. "Seed-time and harvest, summer and winter, day and night, have not ceased." The sun still shines, and the rain falls; the sea carries our ships on its bosom and gives to the fisherman its harvest. We have knowledge which the ancients did not possess, and power over nature they would have thought impossible. We are enlightened, progressive, victorious. What more would we have? Sadness is fatal to sound health, and the want of health increases sadness. It is a doctor's question as much as a religious teacher's. The modern doctor takes cognisance of the whole man: he thinks it his duty to try to "minister to a mind diseased." Indeed, such is the mental worry of the present period, that the doctor who cannot prescribe, and prescribe wisely, some course of treatment for mental strain is not likely to have many patients among those who bear the responsibilities of the world's work. Contentment is a fine simple, like rhubarb; hope is a brave tonic, like quinine or iron; merriment is a brisk and bracing stimulant and tonic combined; and he who, feeling broken down by the incessant strain of life, shall submit to a prolonged and well-directed course of these remedies will almost certainly not fail of a cure. We close by repeating the prescription, with the note of warning which follows,—"*A merry heart doeth good like a medicine, but a broken spirit drieth the bones.*"

(*To be continued.*)

